# *Moringa oleifera* flowers: insights into their aroma chemistry, anti-inflammatory, antioxidant, and enzyme inhibitory properties

**DOI:** 10.1186/s12906-024-04579-y

**Published:** 2024-07-26

**Authors:** Nouran M. Fahmy, Shaimaa Fayez, Radwa Wahid Mohamed, Ahmed M. Elissawy, Omayma A. Eldahshan, Gokhan Zengin, Abdel Nasser B. Singab

**Affiliations:** 1 Department of Pharmacognosy, Faculty of Pharmacy, Ain Shams University, Cairo, 11566 Egypt; 2 Department of Biochemistry and Nutrition, Women’s College for Arts Science and Education, Ain Shams University, Cairo, Egypt; 3 Center for Drug Discovery Research and Development, Ain Shams University, Cairo, 11566 Egypt; 4 Department of Biology, Science Faculty, Selcuk University, Konya, 42130 Türkiye

**Keywords:** *Moringa oleifera* flowers, GC-MS, Essential oil, Antioxidant, Anti-inflammatory, Enzyme inhibition

## Abstract

**Background:**

*Moringa oleifera* is a highly nutritious plant widely used in traditional medicine.

**Results:**

The aroma constituents present in the fresh flowers of *M. oleifera versus* the hydrodistilled oil and hexane extract were studied using GC-MS. Aldehydes were the major class detected in the fresh flowers (64.75%) with *E-*2-hexenal being the predominant component constituting > 50%. Alkane hydrocarbons, monoterpenes, and aldehydes constituted > 50% of the hydrodistilled oil, while alkane hydrocarbons exclusively constitute up to 65.48% of the hexane extract with heptacosane being the major component (46.2%). The cytotoxicity of the hexane extract was assessed on RAW 264.7 macrophages using the MTT assay which revealed no significant cytotoxicity at concentrations of 1 µg/mL and displayed IC_50_ value at 398.53 µg/mL as compared to celecoxib (anti-inflammatory drug) with IC_50_ value at 274.55 µg/ml. The hexane extract of *Moringa* flowers displayed good anti-inflammatory activity through suppression of NO, IL-6, and TNF-α in lipopolysaccharide-induced RAW 264.7 macrophages. The total phenolic and flavonoid content in the hexane extract was found to be 12.51 ± 0.28 mg GAE/g extract and 0.16 ± 0.01 mg RuE/g extract, respectively. It displayed moderate antioxidant activity as indicated by the in vitro DPPH, ABTS, CUPRAC, FRAP, and phosphomolybdenum (PBA) assays. No metal chelating properties were observed for the extract. The enzyme inhibitory potential of the hexane extract was evaluated on acetyl- and butyrylcholinesterases (for neuroprotective assessment), α-amylase and α-glucosidase (for antihyperglycemic assessment), and tyrosinase (for dermoprotective assessment) revealing promising results on cholinesterases, tyrosinase, and α-glucosidase.

**Conclusion:**

Our findings suggested that *M. oleifera* leaves can be considered as a multidirectional ingredient for preparing functional applications.

**Graphical abstract:**

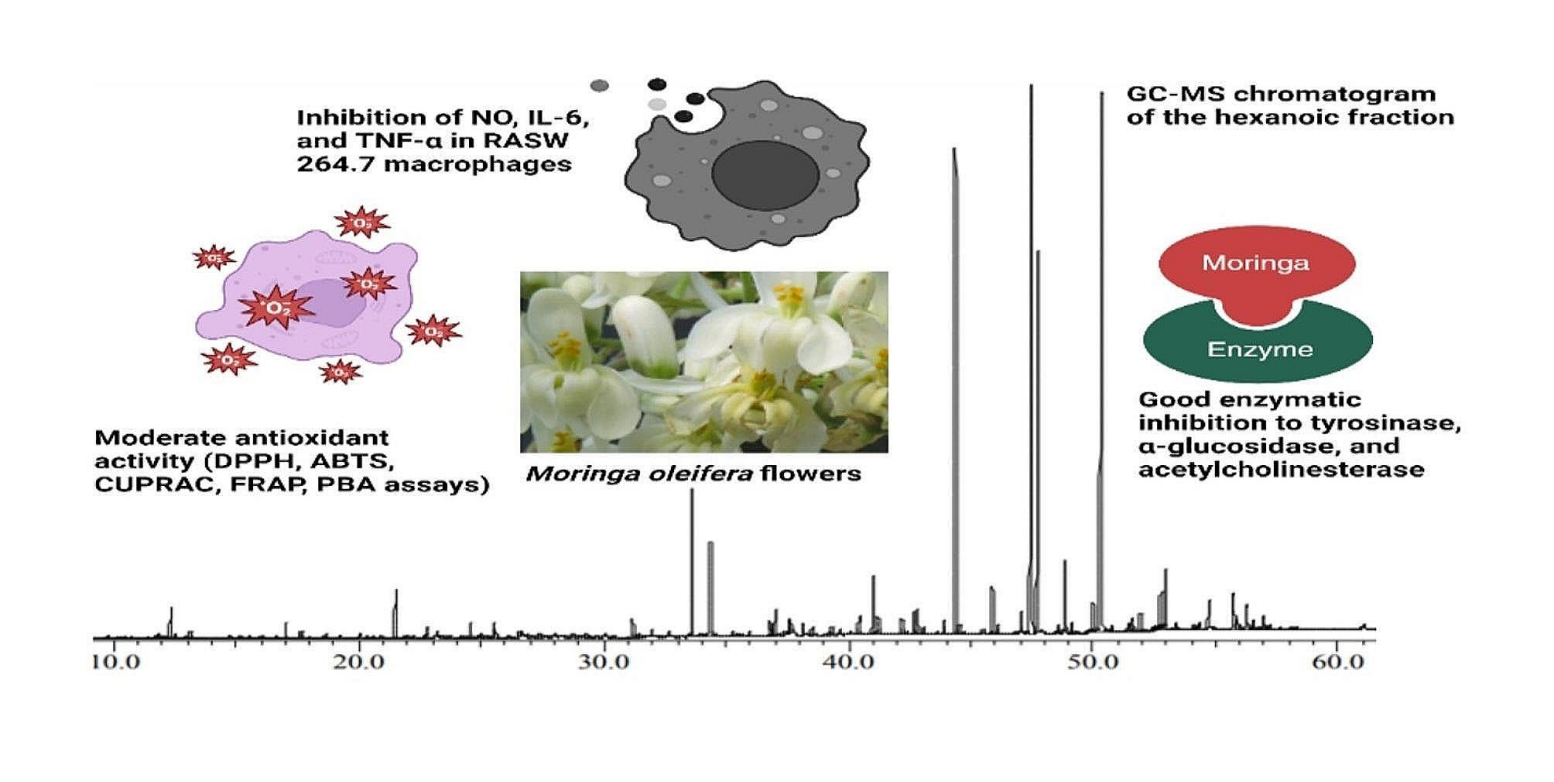

## Introduction

*Moringa oleifera* Lam. (Moringaceae), also known as the drumstick tree, the horseradish tree, and the ben oil tree, is a highly nutritious edible plant whose leaves and immature seed pods are particularly useful as fortificant in food products with a high safety margin [1–3]. The plant is cultivated worldwide owing to its ability to resist severe drought and mild frost conditions [4]. Traditionally, Moringa has been used to treat various health conditions. Its roots were used as a cardiotonic and antiepileptic, while all parts of the plant were utilized to treat high blood pressure, inflammatory diseases, oxidative stress, and infections. The stem bark and flowers were used to regulate blood glucose levels and aid in diabetes management. Leaf extracts are used to treat malnutrition and increase breast milk in nursing mothers as the plant is rich in phytosterols of steroidal nature. Furthermore, the root bark was utilized in the treatment of lipid disorders [2, 4–6]. The coagulant nature of *Moringa* seeds made them of particular use in water treatment applications and biodesel production due to their richness in monounsaturated fatty acids like oleic acid [4, 5]. Phytochemical investigations revealed that the leaves are rich sources of flavonoids, ascorbic acid, carotenoids, minerals, vitamins, glucosinolates, and polyphenolic compounds. The flowers comprise amino acids, flavonoids, traces of alkaloids, and minerals. These classes of compounds contribute to a large extent to its documented medicinal uses and pharmacological activities [4, 5, 7, 8]. Various biological investigations have highlighted the therapeutic potential of Moringa. For instance, the flowers and roots have revealed significant hepatoprotective effects. Antimicrobial activities have been reported for roots, bark, leaves, and flowers extracts. Additionally, extracts from Moringa leaves and seeds have demonstrated promising antitumor and anti-inflammatory activities [2, 8].

The etiology of many diseases is associated with the excessive formation of free radicals that the body can no longer neutralize, leading to oxidative stress. An increase in oxidative stress can lead to cellular, protein, and DNA damage, resulting in various health problems such as aging, inflammation, diabetes, cancer, neurodegenerative and cardiovascular diseases [9–11].

The inflammatory cascade triggered by injury, infection, or tissue damage is a complex process that led to the stimulation of the immune system and the release of key modulators such as nitric oxide (NO) interleukin 6 (IL-6), and tumor necrosis alpha (TNF-α). NO is a signaling molecule that acts as a vasodilator, and is a crucial step in the inflammatory cascade that facilitates the recruitment of immune cells to the site of injury [12]. IL-6 and TNF are pro-inflammatory cytokines essential for the recruitment and activation of immune cells, amplifying the immune response. Dysregulation or excessive production can contribute to chronic inflammatory conditions and various diseases [13].

On the other hand, enzymes in our body are crucial in catalyzing and regulating many biochemical reactions [14]. Cholinesterase is crucial for maintaining nerve response and function. Inhibitors can be used to improve memory function and treat Alzheimer disease through increasing acetylcholine level in the brain and reduction of β amyloid deposition [15]. Tyrosinase catalyzes the conversion of tyrosine into melanin precursors. In skincare, tyrosinase inhibitors are often used to treat hyperpigmentation and protect against the harmful effects of UV radiation [16]. In addition, alpha-glucosidase and alpha-amylase facilitate the conversion of starch into more easily absorbable and simplified sugar forms in the body. Consequently, inhibitors can regulate the postprandial increase in blood sugar levels and help in the management of diabetes [17, 18].

Natural products play a vital role in drug discovery owing to their high chemical diversity and unique biological activities. Many of the top successful drugs in clinical use today are derived from natural sources. Natural products have been used in the treatment of various diseases, including infectious and inflammatory diseases, metabolic and cardiovascular disorders, and neurodegenerative conditions [19, 20].

Extensive research has been conducted on the pharmacological activity of *M. oleifera*, yet comprehensive data on the bioactivity and chemical profile of its flowers remains limited. Given the pivotal role of inflammation and oxidative stress in the development of various chronic and life-threatening conditions, added to the significance of enzymes in regulating biochemical reactions in our bodies, there is a need to explore the antioxidant, anti-inflammatory, and enzymatic inhibitory properties of Moringa flower extracts. In the current work, the effect of the extraction technique on the aroma constituents in the fresh flowers of *M. oleifera* compared to the hydrodistilled oil and hexane extract was studied using GC-MS. Additionally, the antioxidant properties, the potential anti-inflammatory activity using mouse macrophage RAW 246.7 cells (which induces the expression of inflammatory mediators), 1 and the enzyme inhibitory activity on cholinesterase, tyrosinase, alpha-amylase, and glycosidase were investigated.

## Materials and methods

### Plant material and oil extraction

Fresh flowers of *Moringa oleifera* were collected from Faculty of Pharmacy, Ain Shams University, botanical garden in March 2022. The collection of plant material was established in compliance with the national guidelines. Voucher specimens were kept in the Pharmacognosy department herbarium under the code (PHG-P-MO-475). The volatile constituents of *M. oleifera* were characterized using three different extraction techniques namely, headspace, hydrodistillation, and solvent extraction. Headspace technique was employed on 10 g fresh flowers using Shimadzu headspace sampler HS-20 (Kyoto, Japan), oven temperature was set at 80 °C, equilibrating time was 8 min, and pressurizing time was 2.00 min. Hydrodistillation was done on 100 g fresh flowers using a Clevenger system for 4 h [21]. Solvent extraction was done by macerating 200 g of the flowers in *n*-hexane [22]. The obtained oils were weighed, dried, and stored in air-tight amber and sealed vials at -20 °C till use. The yield was calculated as per 100 g fresh flowers (%w/w). The hydrodistilled oil and the *n*-hexane extract yield was 0.005% and 0.25% w/w, respectively.

### GC-MS chromatographic analysis and instrumentation

Gas chromatography coupled to mass spectrometry was carried out utilizing a Shimadzu GC-MS-QP 2010 instrument from Kyoto, Japan, equipped with a DB-1 capillary column (30 m x 0.25 mm i.d. x 0.25 μm film thickness, provided by Restek, USA). n the case of the hydrodistilled oil, the GC run initiated at 45 °C and maintained this temperature for 2 min. Subsequently, the temperature was programmed to increase to 300 °C at a rate of 5 °C/min and held constant for an additional 10 min. The injector temperature was set at 250 °C, and helium served as the carrier gas at a flow rate of 1.41 ml/min. Samples, with a concentration of 1% v/v, were injected with a 15:1 split ratio, and the injection volume was 1 ml. Ionization energy of 70 eV was used with a scan range of 35–500 atomic mass units [23]. For hexane samples, the measurement conditions were the same, except for the GC program. In this case, the initial temperature was set at 50 °C for 3 min, then raised to 300 °C at a rate of 5 °C/min and maintained at 300 °C for 10 min. Compound identification was achieved by comparing the fragmentation pattern of each compound with NIST-17 library. The retention indices (Kovat index) for each compound were calculated after injecting a standard alkane series (C8-C30) under the same method and conditions. These retention indices were compared with the NIST Chemistry Webbook online database and previously reported retention indices of the identified compounds in the literature [22, 24].

### Assessment of the total phenolic and flavonoid contents

The procedure implemented in the determination of the total phenolic and flavonoid contents in the hexane extract of the flowers of *M. oleifera* followed the protocol described by Uysal et al. [25].

### In vitro assessment of antioxidant activities of *M. Oleifera* flower hexane extract

All in vitro antioxidant assays were performed following similar protocol as described by Grochowski et al. [26].The experiments were performed in triplicate and the results were presented as mean and standard deviation.

### Cell culture

Murine macrophages (RAW264.7) were obtained from the American Type Culture Collection (ATCC, VA, USA) and were cultured in Dulbecco’s modified Eagle medium (DMEM supplemented with high glucose) (Invitrogen/Life Technologies) containing 10% v/v fetal bovine serum (FBS) (Sigma Aldrich Co., St. Louis, MO), penicillin (100 U/mL), and streptomycin (100 µg/mL) (Wako Pure Chemical Industries, Ltd., Osaka, Japan) in a humidified atmosphere with 5% CO_2_ at 37 °C. Some cells were sub-cultured for the MTT assay after trypsinization (0.25% trypsin-ethylenediaminetetraacetic acid) (Sigma Aldrich Co., St. Louis, MO), the others were used to measure the production of inflammatory or pre-inflammatory markers such as NO, IL-6, and TNF-α, by addition of *M. oleifera* hexane extract and celecoxib (anti-inflammatory drug).

### MTT assay

*Moringa* hexane extract was dissolved in the least amount of DMSO and subsequently diluted in the culture medium to create the stock solution. RAW264 were cultured in 96-well plates (1.2–1.8 × 10,000 cells/well) in 100 µl growth medium. These cells were then incubated with varying concentrations of *Moringa* extract (1, 4, 16, 63, 250, 1000 µg/mL) for 24 h. DMSO was used as a solvent control. One hundred microliter of 0.5 mg/mL 3-(4,5-dimethylthiaxolone-2-yl)-2,5-diphenyl tetrazoliumbromide (MTT) (Sigma Aldrich Co., St. Louis, MO) solution was added to each well, and plates were incubated for another 2 h at 37 °C. Medium was removed, 50 µL of dimethyl sulfoxide (Invitrogen/Life Technologies) was added to each well to solubilize formazan crystals and absorbance was then measured at 595 nm (iMark, BioRad, Hercules, CA).

### Nitric oxide assay

RAW264 cells were treated with 1 ug/mL of *M. oleifera* hexane extract and celecoxib drug for 24 h. After incubation, the supernatant was collected and used for NO assay. In the case of the NO assay, the supernatants were mixed with Griess reagent containing equal volumes of 1% sulfanilamide (Sigma Aldrich Co., St. Louis, MO) in 2.5% phosphoric acid and 0.1% naphthylethylenediamine dihydrochloride (Sigma Aldrich Co., St. Louis, MO) solution and then incubated at room temperature for 10 min. The concentration of nitric oxide was measured by OD at 595 nm. Sodium nitrite (NaNO_2_) (Sigma Aldrich Co., St. Louis, MO) was used as a standard regent.

### Assessment of the inflammatory mediators IL-6 and TNF-α

RAW264 cells were treated with 1 ug/mL of *M. oleifera* hexane extract and celecoxib drug for 24 h. After incubation, the supernatant was collected and used for cytokine assays. The determination of cytokine concentrations was measured using a Quantikine^®^ enzyme-linked immunosorbent assay kit (R&D Systems, Inc., Minneapolis, MN) for IL-6 and TNF-α following the manufacturer’s instructions.

### In vitro evaluation of the enzyme inhibition ability of *M. Oleifera* flowers hexane extract

The in vitro enzyme-inhibitory assays were all performed as described by Grochowski et al. [26]. The experiments were performed in triplicate and the results were presented as mean and standard deviation **2.10. **.

### Statistical analysis

Data were analysed using the Statistical Package for Social Science (SPSS) version 16.0. Statistical differences between groups were performed using one-way analysis of variance (ANOVA). Values are presented as mean ± standard deviation (SD). The mean difference is considered significant at (*p* < 0.05).

## Results

### GC-MS investigations on the flower volatiles of *Moringa oleifera*

**Fig. 1:**
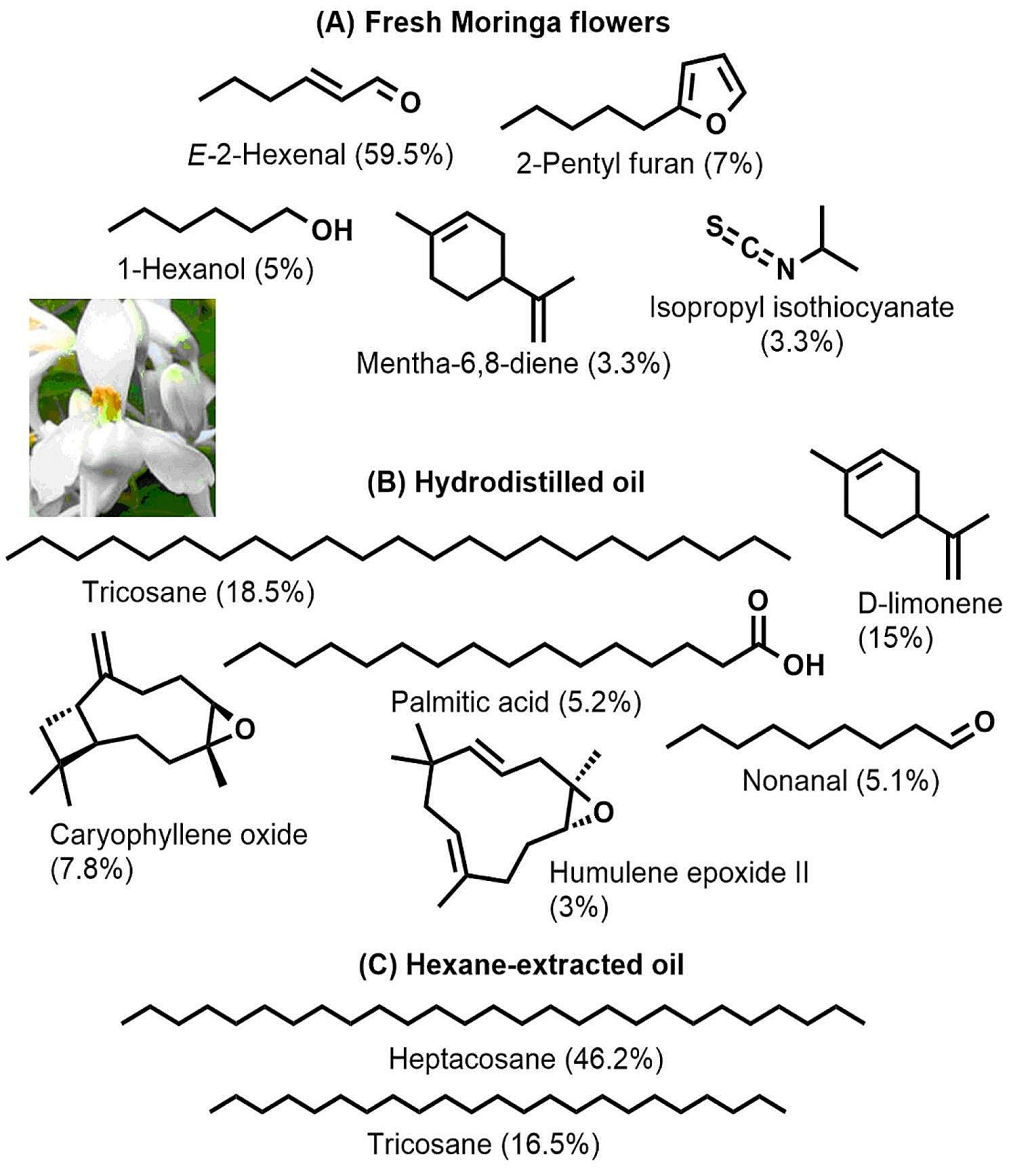
The structures and percentage of major volatiles detected in the GC-MS chromatograms of (**A**) fresh *Moringa* flowers by headspace and processed flowers by (**B**) hydrodistillation and (**C**) hexane-extraction

**Fig. 2:**
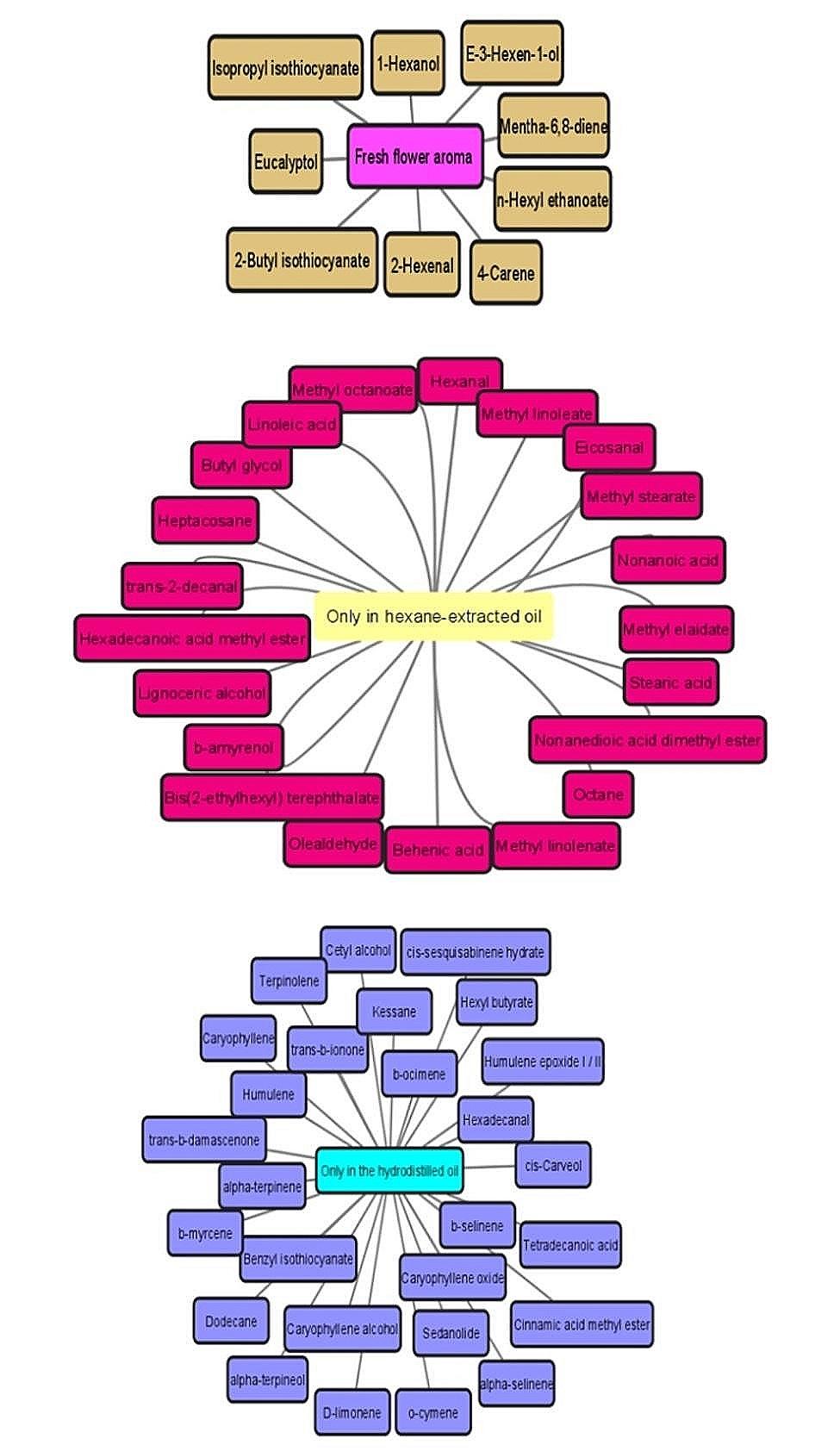
Schematic representation of the metabolites detected exclusively in fresh *Moringa* flowers, in hexane extracted *Moringa*, and in the hydrodistilled *Moringa* oil

Detailed analysis of the metabolites responsible for the aroma of freshly obtained *Moringa* flowers were studied using GC-MS headspace (Table 1). The oil was further extracted by hydrodistillation and hexane, then the volatiles were similarly analyzed by GC-MS. Differences were observed in the aroma constituents between the fresh and processed flowers (Figs. 1 and 2). *E*-2-Hexenal is an aldehyde compound mostly produced by stressed plants and it is the major metabolite detected in the fresh flowers representing ca. 59.5%. Other less relevant compounds were 2-pentylfuran (7%), 1-hexanol (5%), mentha-6,8-diene (3.3%), and isopropyl isothiocyanate (3.3%).

Isothiocyanates, which are most probably formed by the decomposition of the co-occurring glucosinolates, were only observed in the fresh flowers. In the processed flowers, the oil was dominated by alkanes with tricosane being the major constituent in the hydrodistilled oil (18.5%) while heptacosane being the dominant component in the hexane extract (46.2%). The main metabolites in the fresh and processed flowers are presented in Fig. 1. D-limonene constituted ca. 15% of the GC-MS chromatogram of the hydrodistilled oil yet was totally undetected in the hexane extract. Figure 2 explains the differences in the secondary constituents present in the different oils. It was found that 21 metabolites were solely detected in the hexane extract, 28 metabolites were observed only in the hydrodistilled oil, and 10 components were detected only in the aroma of the fresh flowers. These results prove that processing of the flowers by hydrodistillation or extraction significantly affects the aroma of *Moringa* flowers.

### Assessment of the total phenolic and flavonoid content in *Moringa oleifera* flower hexane extract

The total phenolic content in the hexane extract of *M. oleifera* flowers was 12.51 ± 0.28 mg gallic acid equivalent / g extract whereas the total flavonoid was calculated as 0.16 ± 0.01 mg rutin equivalent / g extract. These data indicate the relatively lower proportion of flavonoids in the total phenolic content of the hexane extract.

### Assessment of the antioxidant properties of *Moringa oleifera* flower hexane extract

Antioxidant compounds have the ability to scavenge and, hence, neutralize free radicals owing to the presence of either a phenolic moiety or a conjugated system. The antioxidant activities of the hexane extract of the flowers of *M. oleifera* were quantified as mg trolox equivalent / g extract for DPPH, ABTS, CUPRAC, and FRAP assays with values at 2.71 ± 0.20, 2.34 ± 0.33, 32.61 ± 1.22, and 20.29 ± 0.60, respectively. No metal chelating abilities were observed for the hexane extract. The phosphomolybdenum assay on the hexane extract showed a value of 0.46 ± 0.03 mmol trolox equivalent / g extract.

**Table 1:** Analysis of the aromatic components present in *Moringa oleifera* flowers using GC-MS headspace, GC-MS of volatiles obtained by hydrodistillation, and GC-MS of the *n*-hexane extract

Peak no.	t_*R*_ (min)	KI_exp_	KI_rep_	Identification	Molecular formula	Relative area (%)			Chemical class
						Headspace	Hydrodistilled oil	*n*-hexane extract	
1.	3.58	782	782	Hexanal	C_6_H_12_O	-----	-----	0.37	Saturated aldehyde
2.	4.03	800	800	Octane	C_8_H_18_	-----	-----	0.23	Saturated Alkane
3.	4.43	810	835	Isopropyl isothiocyanate	C_4_H_7_NS	3.29	-----	-----	Isothiocyanate
4.	4.77	823	824	2-Hexenal	C_6_H_10_O	1.52	-----	-----	Unsaturated aldehyde
5.	4.90	827	827	(*E*)-2-Hexenal	C_6_H_10_O	59.53	-----	-----	
6.	5.24	840	840	(*E*)-3-Hexen-1-ol	C_6_H_12_O	0.73	-----	-----	Unsaturated alcohol
7.	5.52	850	850	(*Z*)-2-Hexen-1-ol	C_6_H_12_O	1.15	-----	-----	
8.	5.61	853	853	1-Hexanol	C_6_H_14_O	5.07	-----	-----	Saturated alcohol
9.	6.24	885	884	Butyl glycol	C_6_H_14_O_2_	-----	-----	0.24	Ether derivative
10.	6.87	899	904	2-Butyl isothiocyanate	C_5_H_9_NS	1.06	-----	-----	Isothiocyanate
11.	7.65	927	927	*α*-Pinene	C_10_H_16_	2.85	0.29	-----	Cyclic alkanes
12.	8.72	966	966	*β*-Phellandrene	C_10_H_16_	1.54	-----	-----	
13.	8.81	969	969	*β-*Pinene	C_10_H_16_	1.01	0.23	-----	
14.	9.17	983	983	2-Pentylfuran	C_9_H_14_O	7.04	1.63	-----	Furan derivative
15.	9.33	988	988	*β*-Myrcene	C_10_H_16_	-----	1.86	-----	Unsaturated alkanes
16.	9.39	991	985	*trans*-2-(2-pentenyl)furan	C_9_H_12_O	0.70	0.35	-----	Furan derivative
17.	9.72	1002	1002	*n*-Hexyl ethanoate	C_8_H_16_O_2_	0.74	-----	-----	Ester derivative
18.	10.03	1012	1017	(+)-4-Carene	C_10_H_16_	0.65	-----	-----	Cyclic alkanes
19.	10.06	1013	1013	α-Terpinene	C_10_H_16_	-----	0.20	-----	
20.	10.14	1016	1016	*o*-Cymene	C_10_H_14_	-----	0.46	-----	Aromatic compound
21.	10.35	1022	1022	Eucalyptol	C_10_H_18_O	0.99	-----	-----	Bicyclic ether
22.	10.41	1025	1024	*m*-Mentha-6,8-diene	C_10_H_16_	3.38	-----	-----	Cyclic alkanes
23.	10.45	1026	1026	D-Limonene	C_10_H_16_	-----	15.04	-----	
24.	10.70	1034	1034	*trans*-*β*-Ocimene	C_10_H_16_	-----	0.62	-----	Unsaturated alkane
25.	11.04	1045	1045	*cis*-*β*-Ocimene	C_10_H_16_	-----	0.24	-----	
26.	11.30	1054	1054	*γ*-Terpinene	C_10_H_16_	1.52	0.67	-----	Cyclic alkane
27.	12.25	1084	1084	Terpinolene	C_10_H_16_	-----	0.22	-----	
28.	12.42	1089	1089	Nonanal	C_9_H_18_O	-----	5.16	0.51	Saturated aldehyde
29.	13.11	1105	1105	Methyl octanoate	C_9_H_18_O_2_	-----	-----	0.13	Fatty acid ester
30.	13.68	1130	1127	*cis*, *trans*-Nona-2,6-dienal	C_9_H_14_O	1.15	0.27	-----	Unsaturated aldehyde
31.	13.97	1139	1138	*trans*-2-Nonenal	C_9_H_16_O	2.55	0.86	-----	
32.	14.72	1163	1163	Terpinen-4-ol	C_10_H_18_O	0.65	0.27	-----	Cyclic alcohol
33.	15.08	1175	1175	α-Terpineol	C_10_H_18_O	-----	0.40	-----	
34.	15.30	1182	1182	Hexyl butyrate	C_10_H_20_O_2_	-----	0.29	-----	Ester derivative
35.	15.65	1193	1200	Dodecane	C_12_H_26_	-----	0.72	-----	Saturated Alkane
36.	15.87	1200	1207	*cis*-Carveol	C_10_H_16_O	-----	0.18	-----	Cyclic alcohol
37.	17.04	1233	1234	*trans*-2-Decenal	C_10_H_18_O	-----	-----	0.30	Unsaturated aldehyde
38.	17.66	1254	1256	Nonanoic acid	C_9_H_18_O_2_	-----	-----	0.22	Fatty acid
39.	19.36	1322	1317	Benzyl Isothiocyanate	C_8_H_7_NS	-----	0.23	-----	Isothiocyanate derivative
40.	20.22	1352	1348	Cinnamic acid methyl ester	C_10_H_10_O_2_	-----	0.48	-----	Ester derivative
41.	20.59	1365	1364	*trans*-*β*-Damascenone	C_13_H_18_O	-----	0.34	-----	Ketone derivative
42.	22.03	1417	1418	Caryophyllene	C_15_H_24_	-----	2.05	-----	Unsaturated alkane
43.	22.87	1450	1450	Humulene	C_15_H_24_	-----	2.51	-----	
44.	23.29	1467	1468	*trans*-*β*-Ionone	C_13_H_20_O	-----	0.21	-----	Ketone derivative
45.	23.70	1483	1483	*β*-Selinene	C_15_H_24_	-----	3.32	-----	Cyclic alkane
46.	23.95	1493	1493	*α*-Selinene	C_15_H_24_	-----	0.32	-----	
47.	24.59	1507	1510	Nonanedioic acid dimethyl ester	C_11_H_20_O_4_	-----	-----	0.27	Ester derivative
48.	24.69	1521	1517	Kessane	C_15_H_26_O	-----	0.38	-----	Cyclic ether
49.	25.62	1557	1557	Caryophyllene alcohol	C_15_H_26_O	-----	0.59	-----	Cyclic alcohol
50.	25.89	1568	1568	Caryophyllene oxide	C_15_H_24_O	-----	7.88	-----	Epoxy derivative
51.	26.23	1582	1586	Humulene epoxide I	C_15_H_24_O	-----	0.30	-----	
52.	26.34	1586	1567	*cis*-Sesquisabinene hydrate	C_15_H_26_O	-----	0.44	-----	Cyclic alcohol
53.	26.49	1592	1593	Humulene epoxide II	C_15_H_24_O	-----	3.03	-----	Epoxy derivative
54.	28.56	1681	-----	Sedanolide	C_12_H_18_O_2_	-----	2.30	-----	Cyclic ester (lactone)
55.	30.07	1746	1740	Tetradecanoic acid	C_14_H_28_O_2_	-----	0.29	-----	Fatty acid
56.	31.27	1798	1798	Hexadecanal	C_16_H_32_O	-----	7.62	-----	Saturated aldehyde
57.	31.97	1832	1830	Hexahydrofarnesyl acetone	C_18_H_36_O	-----	0.58	0.12	Ketone derivative
58.	32.68	1866	1866	Cetyl alcohol	C_16_H_34_O	-----	2.98	-----	Fatty alcohol
59.	33.48	1904	1900	Nonadecane	C_19_H_40_	-----	2.99	1.55	Saturated alkane
60.	33.63	1902	1902	Hexadecanoic acid methyl ester	C_17_H_34_O_2_	-----	-----	2.85	Fatty acid ester
61.	34.43	1950	1950	Palmitic acid	C_16_H_32_O_2_	-----	5.21	3.50	Fatty acid
62.	35.50	2001	1999	Octadecanal	C_18_H_36_O	-----	1.62	2.03	Saturated aldehyde
63.	36.82	2062	2069	Methyl linoleate	C_19_H_34_O_2_	-----	-----	0.28	Fatty acid ester
64.	36.89	2065	2058	Methyl linolenate	C_19_H_32_O_2_	-----	-----	0.20	
65.	37.00	2071	2084	Methyl elaidate	C_19_H_36_O_2_	-----	-----	0.48	
66.	37.57	2100	2104	Methyl stearate	C_19_H_38_O_2_	-----	-----	0.27	
67.	37.65	2105	2110	Olealdehyde	C_18_H_34_O	-----	-----	0.82	Unsaturated aldehyde
68.	38.16	2132	2137	Stearic acid	C_18_H_36_O_2_	-----	-----	0.24	Fatty acid
69.	38.52	2151	2123	Linoleic acid	C_19_H_34_O_2_	-----	-----	0.19	
70.	39.32	2195	2221	Eicosanal	C_20_H_40_O	-----	-----	0.75	Saturated aldehyde
71.	41.04	2305	2300	Tricosane	C_23_H_48_	-----	18.49	16.58	Saturated alkane
72.	42.73	2398	2400	Tetracosane	C_24_H_50_	-----	0.96	0.83	
73.	43.90	2461	-----	Lignoceric alcohol	C_24_H_50_O	-----	-----	1.11	Fatty alcohol
74.	44.53	2499	2502	Behenic acid	C_23_H_46_O_2_	-----	-----	0.17	Fatty acid
75.	47.51	2692	2700	Heptacosane	C_27_H_56_	-----	-----	46.29	Saturated alkane
76.	47.75	2709	2704	1,4-Benzenedicarboxylic acid, bis(2-ethylhexyl) ester	C_24_H_38_O_4_	-----	-----	10.05	Aromatic ester
77.	56.27	3322	3337	β-Amyrenol	C_30_H_50_O	-----	-----	0.84	Triterpene
Total identified compounds (%)						97.12	95.08	91.42	
Monoterpenes (%)						10.95	19.83	-----	
Oxygenated monoterpenes (%)						1.64	1.88	0.3	
Sesquiterpenes (%)						-----	8.2	-----	
Oxygenated sesquiterpenes (%)						-----	13.2	0.12	

t_R_: Retention time, KI_exp_: Kovat index experimental, KI_rep_: Kovat index reported

Identification was done based on comparing the mass spectrum and the experimental Kovat index with the reported data on Nist-17 database (National Institute of Standards and Technology) and with previously published data

### Assessment of the effect of *Moringa oleifera* flower hexane extract on the viability of RAW 264.7 macrophages using MTT assay

**Fig. 3:**
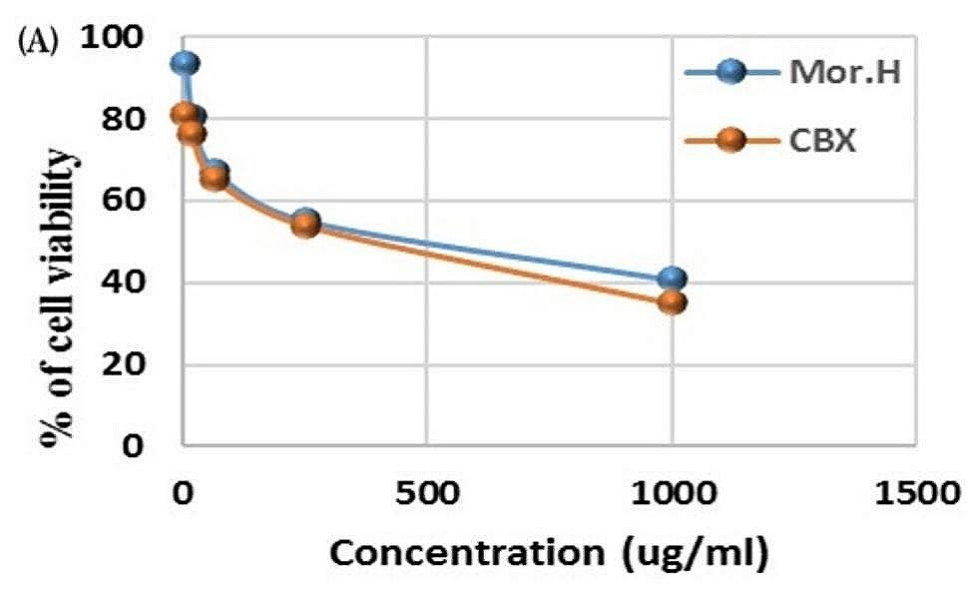
The effect of the hexane extract of the flowers of *M. oleifera* versus celecoxib (standard) on the percentage viability of RAW 264.7 macrophages

Cell viability was assessed using the 3-(4,5-dimethylthiaxolone-2-yl)-2,5-diphenyltetrazolium bromide (MTT) assay. The viability of cells was reduced in a concentration-dependent manner after adding the hexane extract of the *M. oleifera* flowers at concentrations 1, 4, 6, 63, 250, and 1000 µg/mL (Fig. 3). The hexane extract did not show significant cytotoxicity at concentrations of 1 µg/mL and displayed IC_50_ value at 398.53 µg/mL as compared to celecoxib (anti-inflammatory drug), which showed IC_50_ value at 274.55 µg/ml. Based on these results, the hexane extract of the *M. oleifera* flowers was prepared at a concentration of 1 µg/mL and was added to LPS stimulated RAW264.7 macrophages to assess its influence on anti-inflammation.

### Assessment of the effect of *Moringa oleifera* flower hexane extract on nitric oxide (NO), interleukin-6 (IL-6), and tumor necrosis factor alpha (TNF-α) released from RAW264.7 macrophages

**Fig. 4:**
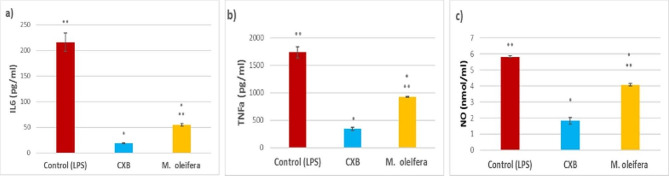
Assessment of (**a**) IL-6; (**b**) TNF-α; and (**c**) NO concentrations after treatment with *M. oleifera* hexane extract and celecoxib standard (CXB). Data are shown as mean ± SD. ٭indicated a significant decrease as compared to the control (lipopolysaccharide LPS) group. ٭٭indicated a significant increase as compared to the CXB group

Figure 4 shows the concentration of NO and the pre-inflammatory mediators IL-6 and TNF-α released from RAW264 macrophages into the medium. *M. oleifera* hexane extract and celecoxib (CXB) were significantly lower (*p* < 0.05) than the control (LPS-RAW 264.7 cells), whereas all the mediator concentrations of *M. oleifera* hexane extract and the control showed a significant increase as compared to the CXB group. These results suggest that the hexane extract of the flowers of *M. oleifera* might show promising anti-inflammatory activity.

### Assessment of the enzyme-inhibitory properties of *Moringa oleifera* flower hexane extract

Assessment of the neuroprotective effects of the hexane extract of *M. oleifera* flowers through suppressing the activity of acetyl- (AChE) and butyrylcholinesterases (BChE) was performed. Results showed that the inhibitory activities of the extract to AChE and BChE were of values 2.55 ± 0.01 and 2.74 ± 0.35 mg galantamine equivalent / g extract, respectively.

The dermo-protective potential of the extract was assessed by measuring its inhibition capacity to tyrosinase, a key enzyme involved in melanin synthesis, which was found to be of value 38.32 ± 0.73 mg equivalent of kojic acid / g extract.

The antihyperglycemic activity of the extract was evaluated by measuring its suppression ability to amylase and glucosidase enzymes involved in carbohydrate digestion. Their inhibition values were 0.40 ± 0.01 and 2.36 ± 0.01 mg acarbose equivalent / g extract for amylase and glucosidase, respectively.

## Discussion

Genus *Moringa* includes 13 species, of which *M. oleifera* is widely cultivated worldwide due to its richness in essential nutrients found in all its parts [27]. GC-MS-assisted studies on fresh flowers, hydrodistilled oil, and hexane extract resulted in the identification of a total of 97.12%, 95.08%, and 91.42% of metabolites, respectively.

Fresh flowers harbour several chemical classes including aldehydes (64.75%), monoterpenes (10.95%), aliphatic and terpene alcohols (8.59%), furan derivatives (7.74%), isothiocyanates (4.36%), and esters (0.74%). The major compound detected in fresh *Moringa* flowers which is eventually responsible for the floral aroma was *E-*2-hexenal, which represented 59.5% of the total GC-MS chromatogram. No fatty acids, alkane hydrocarbons, sesquiterpenes, ketones, or ethers were detected in fresh *Moringa* flowers.

In the hydrodistilled oil of *M. oleifera* flowers, the distribution and relative percentage of chemical classes were different with linear alkanes representing 23.16% followed by monoterpenes (19.83%), aldehydes (15.53%), epoxy derivatives and cyclic ethers (11.59%), sesquiterpenes (8.2%), fatty acids (5.5%), alcohols (4.42%), esters (3.07%), and ketones (1.13%). Tricosane, D-limonene, caryophyllene oxide, nonanal, and palmitic acid constituted 51.6% of *Moringa* hydrodistilled oil. The isothiocyanate content was dramatically reduced in the hydrodistilled oil reaching 0.23% of the total content. The hydrodistilled oil was the only one which contain sesquiterpenes.

In the hexane extracted *Moringa* flowers, alkane hydrocarbons constituted the major class representing ca. 65.48% followed by aliphatic and fatty esters (14.53%), fatty acids (4.32%), aldehydes (3.96%), alcohols (1.35%), and ketones (0.12%). The main fatty acid detected in the hydrodistilled oil and hexane extract was palmitic acid with major predominance in the former. Esters of polyunsaturated fatty acids like linoleic and linolenic acids were exclusively detected in the hexane extract. Furan derivatives, isothiocyanates, mono- and sesquiterpenes along with epoxy and cyclic ethers were absent in the hexane extract.

Having a detailed look at the literature, and to the best of our knowledge, very few data handled the chemical profiling of *Moringa* flowers. Previous GC-MS study on the chloroform fraction of *Moringa* flowers focused mainly on their fatty acid profile with predominance of palmitic acid as the main saturated fatty acid and linoleic and linolenic acids as the main polyunsaturated fatty acids [28]. These data were consistent with that presented in the current study where the aforementioned fatty acids were all observed in the hexane extract, yet only palmitic acid was detected in the hydrodistilled oil. Another study [29] on the hydrodistilled flower oil obtained from *Moringa oleifera* cultivated in Portugal revealed certain consistency with our study in the prevalence of alkane hydrocarbons and palmitic acid in the oil, yet the main hydrocarbons detected in the Portuguese oil were heptacosane, nonacosane, and pentacosane however in the Egyptian oil it was tricosane as the main saturated alkane hydrocarbon detected. The hydrodistilled oil of *Moringa* flowers cultivated in Cuba [30] showed significant differences in the relative percentage of aroma constituents where the Havanan oil contains *E-*nerolidol (13.4%), α-terpineol (7.8%), benzyl isothiocyanate (6.4%), *Z-*3-hexenol (5.7%), 2-methylbutan-1-ol (5.6%), limonene (5.4%), linalool (4.1%), nonanal (4.1%), isopropyl isothiocyanate (3.9%), hexanal (2.4%), *E-*2-hexenal (2.4%), and *E-*2-hexenol (2.3%). In Egyptian hydrodistilled oil, *E-*nerolidol was not observed. Isopropyl isothiocyanate, *E-*3-hexenol, *Z-*2-hexenol, and *E-*2-hexenal were only detected in the fresh, rather than the hydrodistilled, flowers. Limonene content constituted up to 15.04% (rather than 5.4% as observed in the Havanan oil) while α-terpineol and benzyl isothiocyanate (0.23%) were only present in traces (0.40%) in the Egyptian oil. These differences could be due to climatic variations, soil properties, and agricultural conditions which may contribute to changes in the relative percentage of chemical aroma constituents produced by the plant.

Several medicinal and pharmacological effects have been reported on *Moringa oleifera* in particular on the leaves and roots, but comprehensive data on the bioactivity of the flowers are still lacking. As inflammation is a key player that mediate the pathogenesis of many chronic, and sometimes lethal, diseases like cancer, diabetes, cardiovascular, and autoimmune disorders, it is worthy to investigate the anti-inflammatory properties of the flower extract specially since other *Moringa* parts have been shown to display potent anti-inflammatory activity in vitro and in vivo [31–38] which might be attributed to their content of phenolics [39], flavonoids, peptides [40], polysaccharides [41], and/or isothiocyanates [42].

The total phenolic content in the hexane extract of *Moringa* flowers was 12.51 ± 0.28 mg GAE/g extract. This value is actually 3x higher than the total phenolic content in the hexane extract of the leaves which was reported to be 3.85 ± 0.60 mg GAE/g extract [35]. The hexane extract of *Moringa* flowers collected from India showed total phenolic content of 33.08 ± 0.1 mg GAE/g extract which was higher than that in the aqueous and methanol extracts yet lower than the acetone and ethyl acetate fractions [43]. However, the total flavonoid content in the hexane extract of *Moringa* flowers was found to be significantly low reaching 0.16 ± 0.01 mg RuE/g extract compared to the total flavonoid content in the hexane extract of the leaves which was reported to be 2.83 ± 0.74 mg catechin equivalent/g extract [35]. Consistent with our study, the total flavonoid content reported in the hexane extract of the Indian *Moringa* flowers was the lowest (at value 2.31 ± 0.15 mg catechin equivalent/g) compared to ethyl acetate or methanol extracts [43]. In general, the total phenolic and flavonoid content in polar extracts like alcohol or water is much higher than non-polar solvents like ether and hexane [35].

The antioxidant properties of *Moringa* flowers hexane extract were assessed in vitro using DPPH, ABTS, CUPRAC, FRAP, metal chelating assay, and phosphomolybdenum assay. The extract displayed activity corresponding to 2.71 ± 0.2 mg trolox equivalent (for DPPH), 2.34 ± 0.3 mg TE/g (for ABTS), 32.61 ± 1.2 mg TE/g (for CUPRAC), and 20.2 ± 0.6 mg TE/g (for FRAP). Compared to the reported data on the DPPH (from 29 to 35 µM TE/g) and ABTS assays (from 7 to 29 µM TE/g) on the polar (i.e., methanol and water) extracts of *Moringa oleifera* seeds [44], the hexane fraction of *M. oleifera* flowers showed low antioxidant potential owing to its very low content of flavonoids. Despite previous reports on the metal chelating properties of the polyphenol rich fraction of *M. oleifera* leaves [45], we did not observe any metal chelating effect for the hexane extract of the flowers.

The effect of *Moringa* hexane extract on suppressing the release of nitric oxide (a signalling molecule that plays a key role in the pathogenesis of inflammation), interleukin-6 (a proinflammatory cytokine), and tumor necrosis factor alpha (an inflammatory cytokine produced by macrophages during acute inflammation) was assessed in lipopolysaccharide-induced RAW 264.7 macrophages. Compared to the control (LPS- stimulated murine macrophages), the *Moringa* hexane extract and celecoxib reduced NO production significantly by 30% and 68.6% respectively. Furthermore, *Moringa* hexane extract and celecoxib reduced the release of IL-6 significantly by 46.8% and 80.2% respectively, whereas *Moringa* hexane extract and celecoxib reduced the release of TNF-α significantly by 74.7% and 91.3% respectively. Despite the higher efficacy of the non-steroidal anti-inflammatory drug celecoxib than *Moringa* extract, still the latter display higher safety margin with moderate anti-inflammatory effect. Our results are consistent with those previously described on the anti-inflammatory properties of *Moringa* flowers, yet these studies were conducted on the alcoholic extract [46, 47].

Several reports have described enzyme inhibitory potential for *Moringa* although the majority of studies were conducted on the leaves [48–57]. Enzymes involved in carbohydrate digestion like α-amylase and α-glucosidase have been reported to be inhibited by *Moringa oleifera* leaves [53] and seeds [58], also the leaves could inhibit pancreatic cholesterol esterase, which is involved in the control of blood lipids [48]. The hexane extract of *Moringa* roots have been reported to display optimal α-glucosidase and α-amylase which might justify its efficacy as antihyperglycemic remedy [59]. These results are consistent with that presented in the current study about the inhibitory potential of the hexane extract of *Moringa* flowers on α-amylase and α-glucosidase yet with greater potential to inhibit the later enzyme. The hexane extract of *Moringa* flowers displayed neuroprotective potential in patients with Alzheimer due to its inhibition to acetylcholinesterase involved in the breakdown of the neurotransmitter acetylcholine. The ability of the hexane extract of *Moring*a flowers to inhibit tyrosinase, a key enzyme involved in melanin synthesis, make it of potential use in the management of skin depigmentation disorders and could be incorporated among the constituents of a natural remedy for skin whitening. Tyrosinase exhibits a dual role in Parkinson’s disease, serving both as a catalyst for dopamine synthesis crucial for neurotransmission and as a catalyst for the conversion of dopamine to reactive oxygen species (ROS) and toxic metabolites. This process contributes to oxidative stress and neurodegeneration. Thus, its inhibition can also be considered as a neuroprotective possibility [60].

Previous studies showed that undecanoic acid produced by *Aspergillus flavus* (an endophyte on *M. oleifera*) inhibit to high extent tyrosinase enzyme [61]. Another study showed that luteolin might be the major contributor to tyrosinase inhibition as confirmed by its inhibition kinetics [57].

## Conclusion

The aroma of fresh *Moringa oleifera* flowers is mainly attributed to its content of *E-*2-hexenal which constitute more than 50% of its total GC-MS chromatogram. Meanwhile the aroma of the hydrodistilled oil might be attributed to its D-limonene content. Hexane extract is dominated with alkane hydrocarbons like heptacosane and tricosane. Cell viability assay showed that *M. oleifera* hexane extract is not cytotoxic to RAW 264.7 macrophages at concentrations of 1 µg/mL *Moringa oleifera* flowers hexane extract reduced the production of NO as well as the proinflammatory mediators IL-6 and TNF-α in LPS-stimulated RAW 264.7 macrophages. The extract displayed moderate antioxidant activity yet good enzymatic inhibition especially to acetylcholinesterase, tyrosinase, and α-glucosidase. The good anti-inflammatory and enzyme inhibitory properties of the hexane extract of *M. oleifera* flowers beside its non-cytotoxic nature to cells warrant further investigations to explore the extract’s mechanisms of action and potential therapeutic applications.

## Data Availability

Data are available upon reasonable request from the first author; Nouran_Fahmy@pharma.asu.edu.eg.
